# Assessment of lung density in pediatric patients using three-dimensional ultrashort echo-time and four-dimensional zero echo-time sequences

**DOI:** 10.1007/s11604-022-01258-1

**Published:** 2022-03-03

**Authors:** Konstantinos G. Zeimpekis, Christian J. Kellenberger, Julia Geiger

**Affiliations:** 1 Department of Nuclear Medicine, Inselspital, Bern University Hospital, University of Bern, Freiburgstrasse 18, 3010 Bern, Switzerland; 2 Department of Diagnostic Imaging, University Children’s Hospital Zurich, Steinwiesstrasse 75, 8032 Zurich, Switzerland; 3 Children’s Research Center, University Children’s Hospital Zurich, Zurich, Switzerland

**Keywords:** MRI, UTE, ZTE, Lung density, Pediatric

## Abstract

**Purpose:**

Lung magnetic resonance imaging (MRI) using conventional sequences is limited due to strong signal loss by susceptibility effects of aerated lung. Our aim is to assess lung signal intensity in children on ultrashort echo-time (UTE) and zero echo-time (ZTE) sequences. We hypothesize that lung signal intensity can be correlated to lung physical density.

**Materials and methods:**

Lung MRI was performed in 17 children with morphologically normal lungs (median age: 4.7 years, range 15 days to 17 years). Both lungs were manually segmented in UTE and ZTE images and the average signal intensities were extracted. Lung-to-background signal ratios (LBR) were compared for both sequences and between both patient groups using non-parametric tests and correlation analysis. Anatomical region-of-interest (ROI) analysis was performed for the normal cohort for assessment of the anteroposterior lung gradient.

**Results:**

There was no significant difference between LBR of normal lungs using UTE and ZTE (*p* < 0.05). Both sequences revealed a LBR age-dependency with a high negative correlation for UTE (R_s_ =  – 0.77; range 2.98–1.41) and ZTE (*R*_s_ =  – 0.82; range 2.66–1.38)). Signal-to-noise (SNR) and contrast-to-noise ratios (CNR) were age-dependent for both sequences. SNR was higher for children up to 2 years old with 3D UTE Cones while for the rest it was higher with 4D ZTE. CNR was similar for both sequences. Posterior lung areas exhibited higher signal intensity compared to anterior ones (UTE 9.4% and ZTE 12% higher), both with high correlation coefficients (*R*^2^_UTE_ = 0.94, *R*^2^_ZTE_ = 0.97).

**Conclusion:**

The ZTE sequence can measure signal intensity similarly to UTE in pediatric patients. Both sequences reveal an age- and gravity-dependency of LBR.

## Introduction

Magnetic resonance imaging (MRI) for assessment of lung parenchyma used to be challenging and its clinical application in children was limited in the past. Computed tomography (CT) was considered the modality of choice for the evaluation of lung parenchyma and density. However, MRI has been becoming more popular in pediatric exams of lung pathologies and airways in recent years thanks to technical advances and emergence of new sequences [1–8]. In addition, MRI provides an important advantage in avoiding radiation exposure in children, who are the most radiosensitive [9], thus establishing it as safer compared to CT for follow-up scans of pediatric patients [10].

Ultrashort echo-time and zero echo-time sequences are designed to capture the rapid decaying T2* signal of tissues like the lung or bone. Both sequences have been implemented for lung morphology and pathology [11–16], and ultrashort echo-time sequences have been recently used for COVID-19 patients [17–19].

The difficulty in lung MR imaging mainly originates from its physiology. The lung has a 10 times lower proton density than that of other organs, and the T2* relaxation time of the lung parenchyma is extremely short (1.43 ± 0.41 ms at 1.5-Tesla) [20]. Furthermore, the multiple air–tissue interfaces at the level of the alveoli microstructures give rise to strong susceptibility effects that accelerate the signal decay. In this regard, these novel sequences are advantageous in detecting the fast-decaying lung signal in comparison to the conventional spin-echo or gradient-echo sequences with longer echo-times.

Three-dimensional ultrashort echo-time Cones (3D UTE Cones) is a sequence which covers the k-space using multiple twisting radial spokes that form conical surfaces. Therefore, it is a non-cartesian data acquisition method. Cones achieves echo times down to 30 µs [21] and thus is an excellent candidate sequence for lung density imaging. 3D UTE Cones provides higher signal-to-noise ratio than the plain projection reconstruction because it samples the k-space more efficiently and leads to shorter scan times as well. The length of the spokes is operator-adjusted by adding more twisting. The spokes that are longer, have a longer readout so they cover the k-space faster achieving even shorter scan times [21]. The filling of the k-space is realised through the utilization of many size-varying cones that are made up of a different number of spokes.

Respiratory motion-resolved four-dimensional zero echo-time (4D ZTE) [22] is a zero echo-time sequence that is binning the respiratory cycle into different data sets that can be reconstructed separately, so motion is the fourth dimension. The final number or reconstructions depends on the number of the bins that may vary from 4 up to 8. Zero echo-time can achieve even shorter echo-times, namely, near 0, because it employs a continuously switched-on reading gradient during radiofrequency excitation [23–25]. 4D ZTE is a non-cartesian data acquisition scheme using radial spokes as trajectory to fill the k-space. The scan time of zero echo-time is faster than the ultrashort echo-time due to the gradients being constantly on, leading to shorter repetition times and silent scans, rendering the sequence clinically more favourable.

While there are several studies evaluating the performance of UTE on pediatric patients, e.g., with cystic fibrosis [26–28], studies using ZTE sequences are still quite rare [5].

Previous studies performed lung density measurements in comparison to CT [29–32]. A recent publication assessed age-dependency of lung density in children using an UTE sequence [33], while another one describes the application of the UTE sequence for various lung pathologies in children [34]. There is a preliminary study comparing lung imaging between UTE and ZTE in adults [35]. This study showed that ZTE provided higher SNR for the lung parenchyma compared to UTE.

The aim of this study was to investigate the performance of a 4D ZTE for quantitative assessment of lung-to-background signal intensity ratio in comparison with a 3D UTE in pediatric patients. We hypothesized that the 4D ZTE might also reveal age-dependent lung signal intensities similarly to the 3D UTE sequence.

## Materials and methods

### Patient population

We retrospectively analysed all patients who underwent lung MRI at a 1.5-Tesla scanner at a tertiary university children’s hospital between November 2019 and October 2020. 17 patients (7 females, 10 males, median age of 4.7 years, range 0–17 years) with morphologically normal lungs obtained both 3D UTE Cones and 4D ZTE sequences. The patients were scanned for various reasons: six had cardiac anomalies without affecting the lungs, seven obtained lung MRI in the context of staging for extrapulmonary malignancies, the remaining four had thoracic anomalies, such as pectus carinatum. A vascular ring was ruled out in one of the six patients with suspected cardiac anomalies. Two patients had an isolated atrial septal defect without hemodynamical relevance. One patient had a bicuspid aortic valve, one a tricuspid valve insufficiency due to valve dysplasia and another had undergone Rastelli procedure due to pulmonary atresia in infancy. The included patients and their parents gave consent to retrospective data analysis. The necessary approval by the responsible government ethics committee was granted.

### Imaging techniques

An axial 3D UTE Cones and a 4D ZTE were performed in all included patients. The patients were scanned in supine position on a 1.5-Tesla system (Discovery MR450, GE Healthcare, Waukesha, WI). Both sequences were acquired in free-breathing, with respiratory gating using belts near the diaphragm to capture the physiological motion signal. In addition, our 4D ZTE protocol was selected to segment the whole respiratory cycle into 4 phases (4 bins) that were separately reconstructed. Based on the sharpness of the image quality, that means minimum possible blurring, either the second or the third respiratory bin, which roughly correspond to the end-expiration phase, were used for the analysis.

All patients were scanned with a 32-channel cardiac surface coil (GE Healthcare, Waukesha, WI). 13 controls were sedated during the scans. The acquisition parameters for both sequences are shown in Table 1.

**Table 1:** Acquisition parameters of UTE and ZTE

Parameter	UTE	ZTE
TR (ms)	3.7	294
TE (ms)	0.032	0.016
Spoke readout time (ms)	740	–
Flip angle (degrees)	5	2
FOV (cm^2^)	20 × 20/34 × 34	20 × 20/36 × 36
Slice thickness (mm)	2–3	1.5
In-plane resolution (mm)	0.9	1.0
Acquisition matrix	384 × 384	192 × 192–256 × 256
Receiver BW (kHz)	256	62.5
Acquisition time	3–5 min	~ 3 min

*TR*  repetition time, *TE*  echo-time, *FOV*  Field of view, *BW*  Bandwidth

### Quantitative analysis

**Fig. 1:**
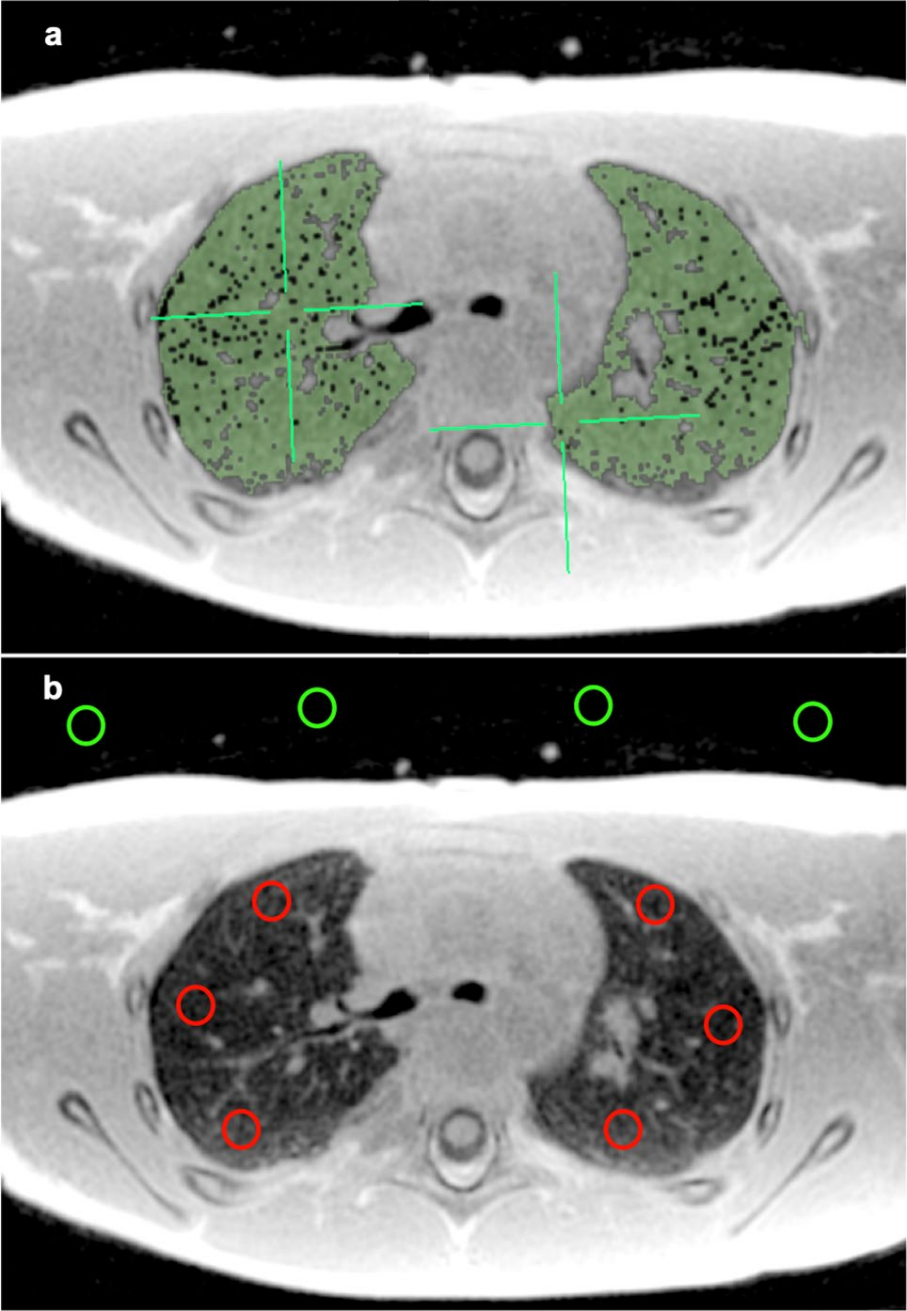
Manual lung segmentation by thresholding and region-of-interest placement of a 6-year-old female subject.** a** Axial slice showing the manual segmentation of lung parenchyma (green). **b** Axial slice with background (green) and lung (red) regions-of-interest

The right and left lungs were manually segmented by signal intensity thresholding avoiding high intensity vessels, and average signal values were extracted (Fig. 1). Lung segmentation was performed using the open-source image analysis software Osirix MD version 11.03 (Pixmeo Sarl 2016, Bernex, Switzerland). Four two-dimensional regions-of-interest (size of 20 mm^2^ each) were drawn in the air anterior to the patients’ thorax in each slice and the average background signal intensity was extracted (Fig. 1). One additional two-dimensional region-of-interest of variable size was drawn inside the trachea on an axial slice at the carina level to measure the signal intensity of the air column within the trachea. In addition, two-dimensional regions-of-interest (size of 20 mm^2^ each) were positioned in the anterior, middle, and posterior anatomical regions of each lung, for both sequences at the level of the carina in the patients with normal lung parenchyma (Fig. 1). The regions-of-interest did not include any high intensity vessels to avoid signal contamination. Finally, the average signal of both lungs was used to determine the whole lung signal intensity while the average anterior signal intensity (respectively middle and posterior) was determined by the average signal of the anterior region-of-interest of both lungs (respectively middle and posterior regions-of-interest).

The lung-to-background ratio (LBR) was calculated as the ratio of the whole lung to background signal intensity. Thus, since both sequences have different scan parameters and physical characteristics, the LBR normalizes the intensity to the respective noise level of each sequence, allowing a reliable inter-sequence comparison.

Image quality parameters such as the signal-to-noise ratio and contrast-to-noise ratio were calculated using the signal from the trachea region-of-interest since it was more reliable for the noise level inside the lungs. The signal-to-noise ratio was defined as the ratio of the lung signal intensity to the standard deviation of the signal intensity of the trachea’s region-of-interest. The contrast-to-noise ratio was calculated as the ratio of the difference of the lung and trachea signal intensities to the standard deviation of signal intensity of the trachea’s region of interest.

### Statistical analysis

Descriptive statistics were used to measure the median value with the corresponding standard deviation to analyse the lung-to-background ratio differences between the cohorts and sequences. Two-tailed Wilcoxon signed ranked test was applied for the intersequence comparison with a *p* value < 0.05. Spearman rank correlation test was applied to evaluate the age dependency of the lung-to-background ratio. Statistical analysis was performed in R (version 3.5.1; R Foundation for Statistical computing, Vienna, Austria).

## Results

**Fig. 2:**
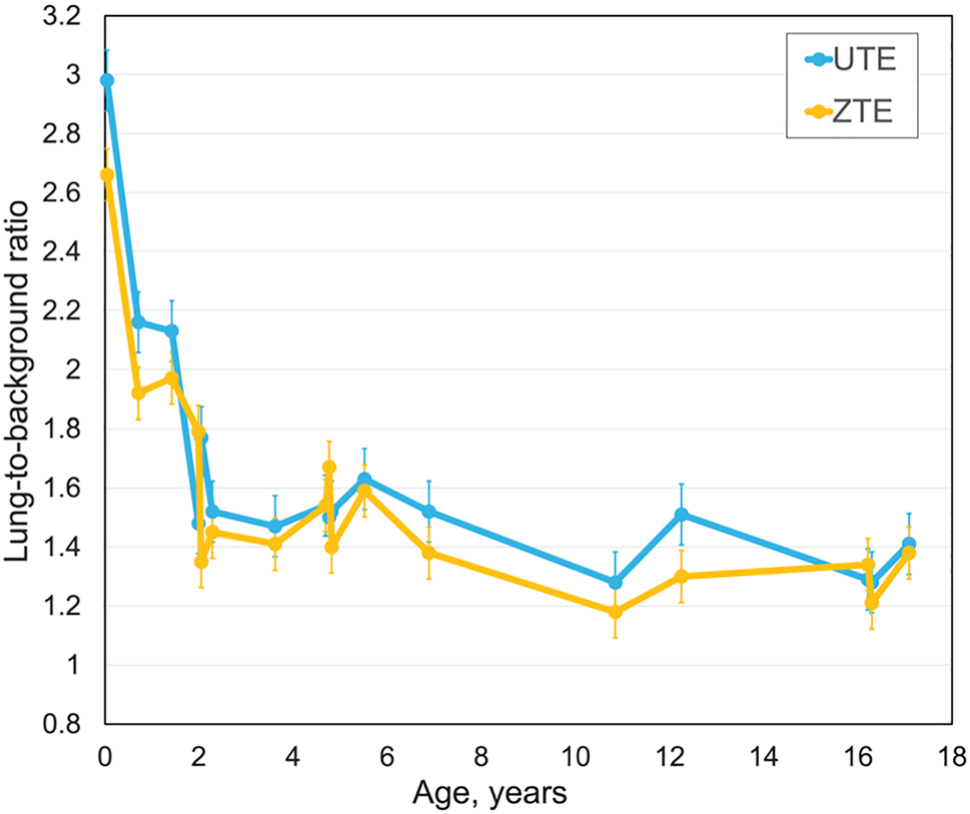
Lung-to-background signal intensity ratio curve depending on age**.** Whole lung-to-background ratio curve, for ultrashort echo-time and zero echo-time for controls related to age

The lung-to-background ratio for patients with normal lungs was higher than 1 for all ages and comparable between the two sequences (Fig. 2). The median value of the lung-to-background ratio for all patients for the ultrashort echo-time sequence was 1.52 $\pm$ 0.41 and for the zero echo-time sequence 1.41 $\pm$ 0.34. Both sequences revealed the highest lung-to-background ratio in newborns with values of 2.98 for the ultrashort echo-time sequence and 2.66 for the zero echo-time sequence. A signal drop was observed for up to two years of age for both sequences (1.77/1.35 respectively). The lung-to-background ratio curve revealed a stable signal plateau for both sequences (range 1.52–1.45 to 1.41–1.38, respectively) between the age of 2 and 17 years. There was no significant difference in lung-to-background ratio between the two sequences (*p* > 0.05).

Both sequences showed a high negative correlation between the lung-to-background ratio and increasing age (Spearman correlation coefficient *R*_s_ =  – 0.77/ – 0.81 for ultrashort echo-time and zero echo-time, respectively; *p* < 0.001).

**Fig. 3:**
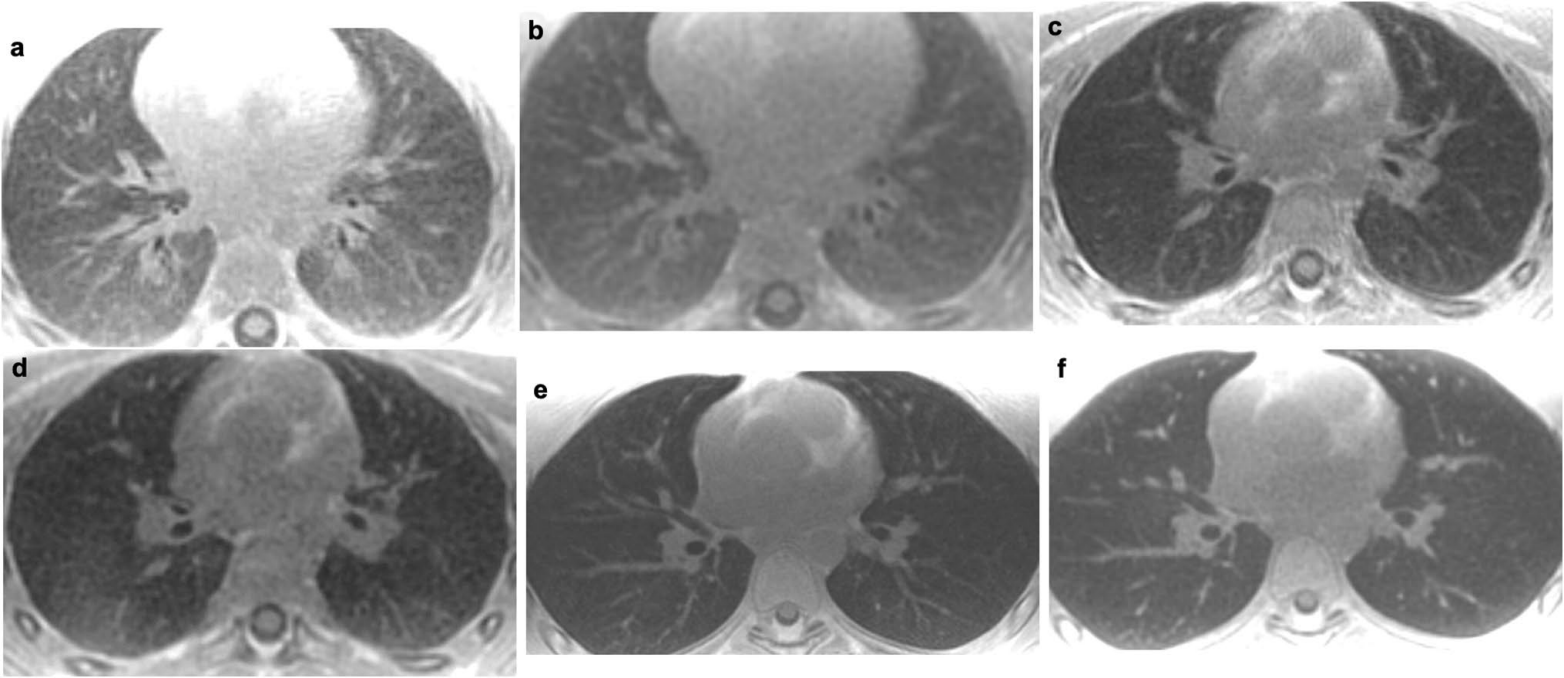
Comparison of the ultrashort echo-time (left column) and zero echo-time sequence (right column) depending on age in patients without lung pathology. **a** Axial slices at the hilum level of a 1-year-old boy and **b**, 4-year-old boy **c** and **d** and 17-year-old teenager **e** and **f** reveal signal change in lung parenchyma between 1 and 4 years of age for both sequences

Qualitatively, both sequences show an increased lung parenchymal signal intensity in young children that decreases with increasing age (Fig. 3).

**Fig. 4:**
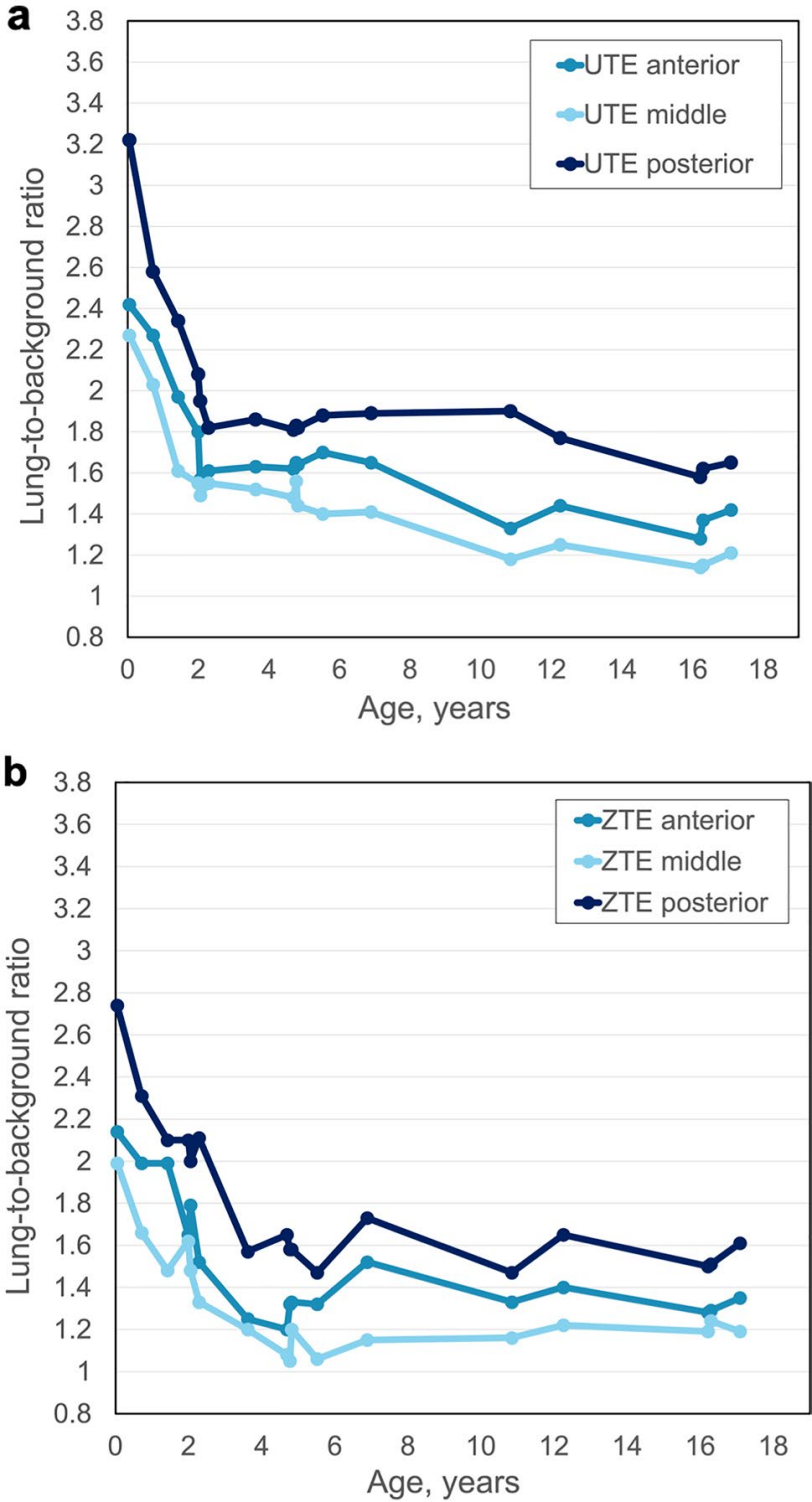
Lung-to-background signal intensity ratio curve depending on age and gravity gradient. Regional lung to background ratio for anterior, middle, and posterior areas for **a** ultrashort echo-time and **b** zero echo-time for controls related to age

The anterior, middle, and posterior lung-to-background ratio curves in relation to patient age follow the same curve as for the whole lung. For both sequences, the posterior lung-to-background ratio was higher compared to the anterior and middle lung-to-background ratio for all ages and was highest for newborns. We found a rapid decrease in lung-to-background ratio until the age of 2 years. The age dependency is shown in Fig. 4a for the ultrashort echo-time sequence and Fig. 4b for the zero echo-time sequence.

**Fig. 5:**
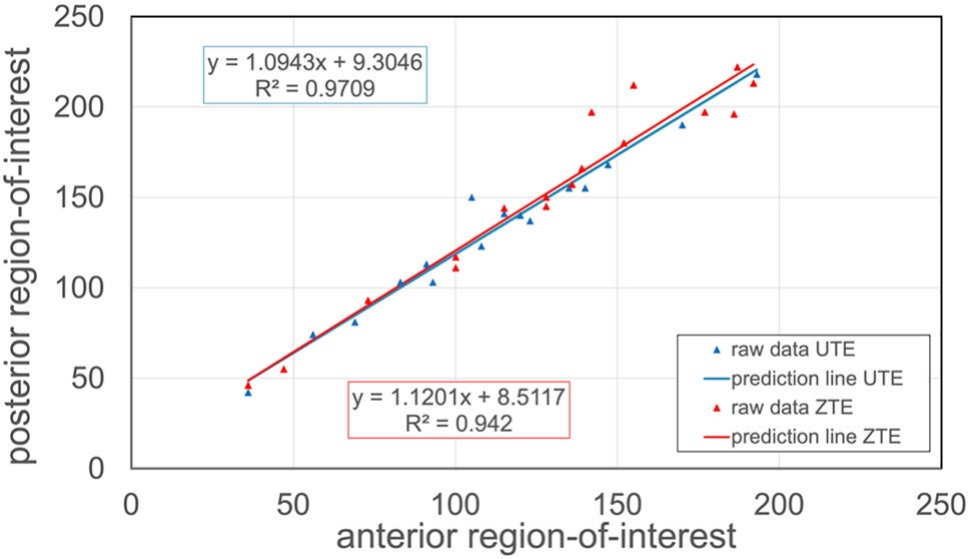
Correlation of anterior to posterior region-of-interest signal intensities for ultrashort echo-time and zero echo-time, for the control cohort for all ages

To determine quantitatively the difference of the posterior and the anterior lung-to-background ratio, a linear regression was applied. Figure 5 shows the correlation of anterior (*x*-axis) and posterior regions-of-interest (*y*-axis) for both sequences for all patients without pulmonary disease. The corresponding correlation coefficients were *R*_1_^2^ = 0.971 and *R*_2_^2^ = 0.942, which showed high correlation of 97% and 94%, respectively. After the linear fitting, the posterior lung-to-background ratio was 9.4% higher than the anterior lung-to-background ratio for the ultrashort echo-time and 12% higher for the zero echo-time sequence.

**Fig. 6:**
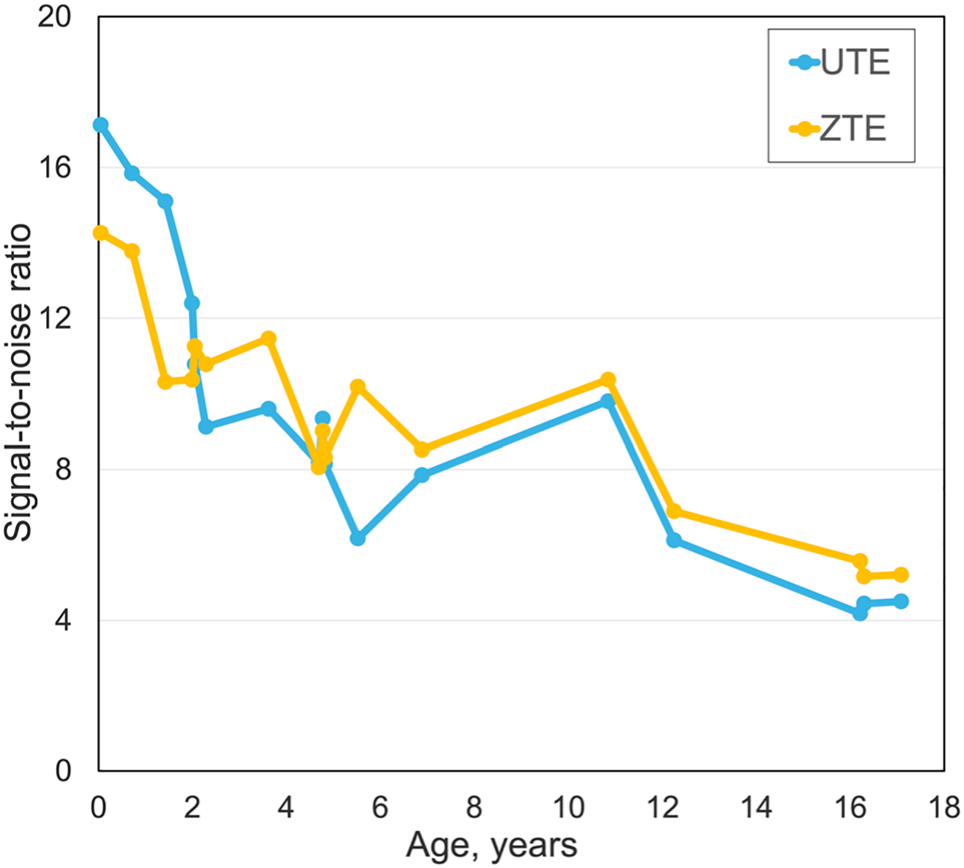
Whole lung signal-to-noise ratio curves, for ultrashort echo-time and zero echo-time, for controls related to age

**Fig. 7:**
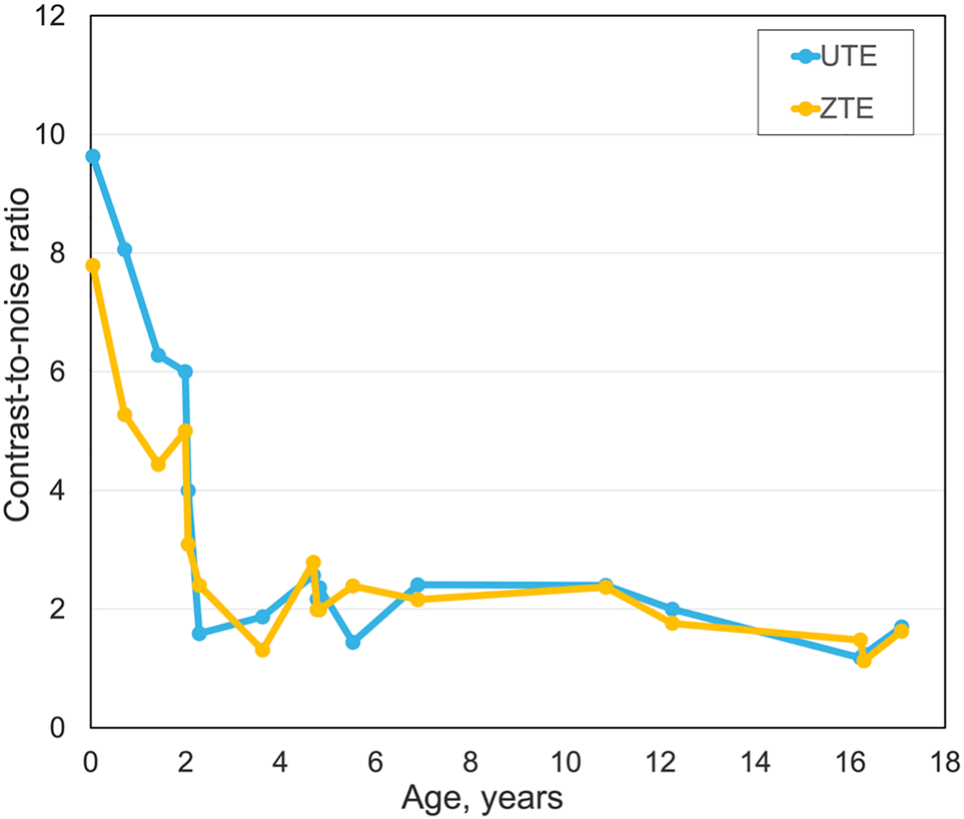
Whole lung to trachea contrast-to-noise ratio curves, for ultrashort echo-time and zero echo-time, for controls related to age

Signal-to-noise ratio and contrast-to-noise ratio curves (Figs. 6 and 7) were comparable and showed no statistically significant difference (*p* > 0.05) for ultrashort echo-time and zero echo-time sequences. Both sequences exhibited the highest values in newborns with a steep decrease to the age of two years. The ultrashort echo-time sequence showed higher values than zero echo-time sequence for newborns while the zero echo-time sequence had higher signal-to-noise ratio values for older patients.

The values of signal-to-noise ratio for ultrashort echo-time and zero echo-time sequences decreased from 17 and 14 in newborns to 4.5 and 5.2 in young adults. Furthermore, the contrast-to-noise ratio varied from 9.6 to 1.7 for the ultrashort echo-time sequence and from 7.8 to 1.6 for the zero echo-time sequence.

## Discussion

We could demonstrate in this study that lung signal intensities can be measured by using a zero echo-time sequence in pediatric patients. The sequence is able to detect age-dependent changes similarly to ultrashort echo-time sequences. Both sequences can visualize lung parenchyma with its rapid signal decay due to its very short T2* time in contrast to conventional MR sequences that are based on proton imaging.

With improvements in MRI hardware and software, particularly with the introduction of non-Cartesian data acquisition schemes, echo times near zero can now be used for image acquisition either as ultrashort echo-time or as zero echo-time acquisitions. Based on their acquisition scheme both sequences are three-dimensional [5]. Zero echo-times sequences have the advantage of minimal gradient switching and, therefore, allow for silent lung imaging compared to UTE. Therefore, ZTE may be more clinically useful and comfortable for the patients supposing that delivers similar results to UTE.

An ultrashort echo-time sequence—the so-called Cones sequence—proved to assess the lung signal intensity decrease from newborns to the age of two years which was consistent with previous CT results [28, 36]. In this study, we were able to demonstrate the same tendency using a zero echo-time sequence that revealed the same signal decrease from newborns to infants. In newborns, the lung signal intensity was three times higher than the background signal using the ultrashort echo-time sequence, whereas the signal intensity measured with the zero echo-time sequence was 2.6 times the background signal. Both sequences had a stable lung-to-background signal intensity ratio through childhood and adolescence until early adulthood with only minor differences concerning median (1.5 vs 1.4) values.

Both sequences revealed an increased posterior lung-to-background ratio compared with the anterior lung-to-background ratio for all patients of the normal cohort, independent of age. These findings are in line with previously described CT results in the literature [36–39]. The zero echo-time sequence had increased posterior-to-anterior values compared to the ultrashort echo-time sequence. This could be caused by noisier signal intensities in the anterior region in the zero echo-time sequence compared to the ultrashort echo-time sequence. The fact that both sequences were able to depict the anteroposterior gravity gradient of the lung density supports our hypothesis of the correlation between the lung signal intensity measured by MRI and the physiological lung parenchymal density. The signal-to-noise ratio and contrast-to-noise ratio of the posterior lung-to-background ratio was higher compared to the anterior and middle lung-to-background ratios for both sequences for all patients. The ultrashort echo-time sequence had higher signal-to-noise and contrast-to-noise ratios for newborns while zero echo-time provided slightly higher signal-to-noise ratio for the older patients. This could probably be caused by lower noise of the ultrashort echo-time sequence in newborns and lower noise of the zero echo-time sequences in older children.

The ultrashort echo-time sequence is a highly sensitive method that may also be valuable for the multiple follow-up imaging examinations required for lung patients during treatment [26]. Both sequences have been investigated in recent studies by Bae et al. [22, 35]. The four-dimensional zero echo-time sequence provided images of lung parenchyma with improved signal-to-noise ratio and contrast-to-noise ratio compared with its three-dimensional variant. In addition, the zero echo-time sequence had higher signal-to-noise and contrast-to-noise ratios than the ultrashort echo-time sequence for adult patients, similarly to our findings.

ZTE may prove to be more patient comfortable compared to UTE due to silent and faster scans while providing similar if not better diagnostic images for general pediatric imaging. UTE may be the better sequence for newborns while delivering also higher contrast for long T2 tissues compared to ZTE.

Limitations of our study are based on variations in detected MRI signal which depend on scan parameters and patients’ properties such as height and weight.

The noise distribution is spatially variable affecting the signal intensity. The signal has a range of values within a certain field of view depending on which area of the body is being imaged. The statistical average of the signal and the noise can be more reliable for the image quality and quantitative evaluation.

The captured signal is proportional to the voxel size. Both sequences had comparable in plane-resolution but different slice thickness. The ultrashort echo-time sequence had a thicker slice thickness and could, therefore, have theoretically between 8% (with 2 mm slice thickness) up to 62% (with 3 mm slice thickness) higher signal. The zero echo-time sequence has shorter echo-times leading to higher signal as well. However, the sequences are different, and thus, other parameters may have an impact on the signal.

Even though the regions-of-interest were drawn at the same anatomical regions, they were not drawn exactly at the same regions due to different patient size and characteristics. Another consideration is the presence of atelectasis due to sedation to a few newborns. In these cases, the regions-of-interest were placed just beside the atelectatic regions to avoid signal intensity contamination. As atelectases have a higher signal, they were cut off during the appropriate intensity thresholding by manual segmentation of the whole lung signal intensity assessment.

Inconsistent respiratory gating during data acquisition due to irregular breathing may lead to further limitations. Lung signal might change, and blurring artifacts might occur in cases of incorrect gating during the end-expiration phase.

The overall patients’ cohort is rather small and heterogeneous regarding age and disease. Unfortunately, we had to exclude several patients in whom at least one of the sequences had poor image quality resulting in a smaller cohort than initially aimed at. However, both sequences were able to demonstrate the age dependent changes of lung signal intensities.

For reasons of radiation exposure, we did not compare our MRI results with CT lung density measurements. However, the lung-to-background ratio curves for both ultrashort echo-time and zero echo-time sequences follow the characteristics of the CT lung density curves in the literature.

Ultrashort and zero echo-time sequences are promising within the scope of modern lung imaging as a radiation free imaging method which is particularly important in children. Both sequences are possible candidates for MR quantification of normal and pathological lung.

## Conclusion

Ultrashort echo-time and zero echo-time sequences are able to assess lung density by capturing higher lung signal intensity in comparison to the background air. Based on their efficiency in visualizing and quantification of lung parenchyma, they may further be clinically utilized for lung MRI in children without exposing the patients to unnecessary radiation exposure.
